# The short-term economic influence analysis of government regulation on railway freight transport in continuous time

**DOI:** 10.1371/journal.pone.0298614

**Published:** 2025-04-10

**Authors:** Jingwei Guo, Jiayi Guo, Tingyue Kuang, Yimin Wang, Wenxin Li

**Affiliations:** 1 Faculty of Business, City University of Macau, Macau, China; 2 School of Energy Science and Engineering, Henan Polytechnic University, Jiaozuo, China; 3 Hubei Key Laboratory of Power System Design and Test for Electrical Vehicle, Hubei University of Arts and Science, Xiangyang, China; Tongji University School of Economics and Management, CHINA

## Abstract

Regulatory impact analysis, a crucial aspect of the development of railway transportation in China, has faced increasing challenges due to the complex nature of the transportation system and various influencing factors. This study proposes an improved comparative static model (ICSM) that intelligently integrates recursive principles and the Laplace transform to estimate the impact of macroeconomic policy reforms on the economic variables of China Railway Corporation (CRC). The ICSM integrates recursive principles and Laplace transform to solve complex conditions of eigenvalues as multiple roots and a coefficient matrix unable to diagonalize in the ICSM. Additionally, the paper discusses the government’s potential impact on variables such as freight and labor after reform and highlights the sensitivity of tax policy compared to investment subsidies in the short term. The findings provide theoretical and methodological support for optimizing railway freight management during the reform process and can serve as a reference for temporary economic policy adjustments.

## 1. Introduction

New policies often guide industrial progress to facilitate the desired future. The railway industry is no exception, and government regulation continues to be improved to ensure industry with healthy development as well as social welfare. During the past three decades or so, China Railway has implemented many policies for reform. The regulatory process in China has changed several times and is still changing and evolving. Especially since the last regulatory reform, the Ministry of Railways has been split, with its regulatory powers going to the Ministry of Transport of the People’s Republic of China (MOT), while its commercial operations will be run by China Railway Corporation (CRC). In other words, China’s government has gradually eased restrictions on rail transport since 2013 [1,2]. Although the Chinese government believes that policy reform helps CRC comply with market rules, satisfy the requirements of transportation demand [3], better serve passenger [4,5] and freight transportation, and be more conducive to its development, it has also brought about certain subsequent controversy in academia: (1) Whether the regulation of the railway industry maximizes social welfare is questionable. (2) The implications of policies reform for current public policy issues in transportation are difficult to predict.

Under a regulated environment, the government considers CRC as a natural monopoly enterprise that might use its monopoly power to raise prices to infringe consumer interests. Therefore, CRC is still fully implemented by the central government and strictly controlled for a long time. In particular, freight rates are regulated to ensure the transportation of materials and prevent blocked transportation caused by excessively high freight rates. The purpose of regulating freight rates is to maximize social welfare. It is undeniable that the freight rate control mechanism has played an important role in socio-economic development and emergency material transportation during China’s Planned Economy period (1950–1992). However, in the current socialist economy with Chinese characteristics (1992–), whether the regulation of the railway industry is reasonable, whether the regulation can improve the efficiency of the whole society has caused much more controversy [6,7]. According to China’s deregulation experience in Posts, Telecommunications, Petroleum, Electricity, and Other Industries, deregulation and the introduction of competition in the public services sector not only increase productivity in this sector but also have a significant spillover effect, indicating that deregulation has not harmed social welfare in the long run. Consequently, rail transport is undergoing a process from the government’s regulation to deregulation.

Few empirical studies directly evaluate the welfare consequences of policy reforms in China, despite their historic prevalence and current popularity. Scholars have focused on predicting social welfare and have found that implementing simple price-cap regulation without addressing congestion issues can lead to efficient railway operations but reduced capacity and lower social welfare [8]. Privatization of the Canadian National Railway has been shown to improve both operational and social welfare outcomes [9]. The impact of price discrimination on welfare in the high-speed railway system varies, with higher welfare observed when the difference in gain from travel outweighs the time value difference between business and leisure passengers [10]. A numerical study on the Chinese market suggests that deregulation can maximize total social welfare if the market is initially inefficient, while regulating activities are inefficient and eventually abandoned for the sake of social objectives and market efficiency.

These studies, both theoretically and empirically, verify the necessity and feasibility of deregulation. However, this research focuses on the impact on long-term social welfare. When thinking about the impact of policy reform, we should not only consider long-term(more than ten year) effects but also a series of short-term(less than one year) changes, including social welfare [11]. In the existing analysis method of short-term welfare, the methods of Turnovsky [12,13], Judd [14], and Chamley [15] played a dominating role. Besides, short-term analysis is particularly important in numerous fields. Gal and Hijzen [16] examined the impact of product market reforms on 10 regulated industries and 18 advanced economies from 1998 to 2013. Khoo et al. [17] used spectral analysis to examine the immediate effects of a policy change on private and public transportation in the Klang Valley region of Malaysia. Li and Kamargianni [18] developed a mixed nested logit model to study short-term car-sharing choice behavior. In addition, Bettocchi et al. [19] developed a micro-founded model to analyze the impact of predicted trends of macroeconomic variables on an indicator to monitor its short-term evolution.

However, the existing research and algorithms have yet to deal with multidimensional systems with many states and many free variables. For this problem, several studies have been done to obtain an easier mathematics model of numerical value solution using different matrix operations, such as pseudoinverse and projection matrices [20], generalized inverse [21], compact density-matrix [22], and Judd matrix [23]. Although these methods and algorithms have promoted the development and application of multidimensional systems to some extent, it is still difficult to solve a matrix that has a negative eigenvalue. In this regard, optimal control models [24], standard models of exogenous and endogenous growth [25,26], and multi-sector perfect foresight models [27,28] are the in-depth studies on the eigenvalue problem. Particularly, Strulik [29] established a new view on evaluating the macroeconomic consequences of temporary tax cuts and expected tax reform. Although these approaches have been successfully applied to different types of eigenvalues, they fail to deal with the issue of the eigenvalue as multiple roots. At the same time, that research has been confined to areas such as tax, international economic aid, fiscal policy, or monetary policy effects on national macroeconomics. Little research on microeconomic characteristics combined with a single industry dabbles in.

To address the shortcomings of previous research, this paper proposes a novel prediction model and algorithm to investigate how variables such as government policies and railway system reform affect transportation economy and social welfare in the short term. Based on the problem of regulatory failure in China Railway, a multidimensional differential dynamical system that conforms to the characteristics of the railway transportation market is proposed. Furthermore, the system is improved to contain four ordinary differential equations for the transportation industry to discuss two different control modes: tax controls and investment subsidies. Due to the matrix calculation problem, a new evaluation method using recursive principle and Laplace transform to solve the condition of eigenvalue as multiple roots and coefficient matrix unable to diagonalize in an improved model is proposed, so as to estimate macroeconomic policy reform impact on the economic variables in the short-term, temporarily adjust economic policy and so on. In addition, our research will also have some implications for the Chinese government’s decision policy in the railway industry in the future. In summary, our main contributions are four-fold and summarized as follows:

a) We introduce a multidimensional differential dynamical system, which conforms to the characteristics of the railway transportation market, to describe the dynamics of the economy with the new fiscal policies in the deregulated railway industry environment.
b) Using economic analysis, we show that the poor performance of railway cargo transportation is ultimately limited by freight rate regulation.
c) In addition, we present an improved solution algorithm that intelligently integrates the recursive principle and the Laplace transform to solve the ICSM condition of eigenvalue as multiple roots and coefficient matrix unable to diagonalize.
d) We analytically and numerically show the feasibility and effectiveness of our proposed algorithm when two different implementation measures are employed.

The rest of this paper is organized as follows. In Section II, we discuss the inefficiency and irrationality of China’s railway regulations and give a detailed description of the problems in the process of reform to be solved. In section III, a multidimensional differential dynamical system containing four ordinary differential equations based on a comparative static model is proposed. Then, we propose a new method to solve the problem of eigenvalue as multiple roots and a coefficient matrix unable to diagonalize in Section IV. A case study and its results are presented in Section V. Finally, the conclusion and discussion are given.

## 2. Correlation analysis of china railway regulatory failure

In order to discuss macroeconomic policies reform impact on the economic variables under deregulation, we mainly concentrate on the regulatory failure in China Railway, discovering the problems in the process of reform to be solved in section III.

### 2.1. Economic description of railway regulation

The government’s role in a modern economic system is crucial. In a western-style democracy, it is widely recognized that large firms with economies of scale seek to gain excessive profits through their monopoly power, leading to the concept of a “natural monopoly”. The CRC, which has natural monopoly features due to the characteristics of large infrastructure construction investment, great sunk costs, and a long investment payback period, is no exception and has been highly regulated since its establishment [30,31]. According to economic theory, it appears reasonable for the government to control and regulate the macroeconomic.But one of the main objectives of putting a particular industry under surveillance is to maximize social welfare.

Every regulatory decision is based on whether it will improve the efficiency of the whole society as opposed to whether it will improve the economic efficiency of the CRC as a whole. On this basis, the railway industry is one of the traditional domains for governmental regulation to prevent profiteering. Governments tend to adopt freight rate controls on railway transportation in China [32]. However, the freight rate regulation did not achieve this goal. Instead, the regulation damaged the enthusiasm of railway transport enterprises operating, disturbed the order of the freight transportation market, disrupted the fair competition of the market economy, and did not play the adjustable role for national resource allocation as it should be.

**Fig 1:**
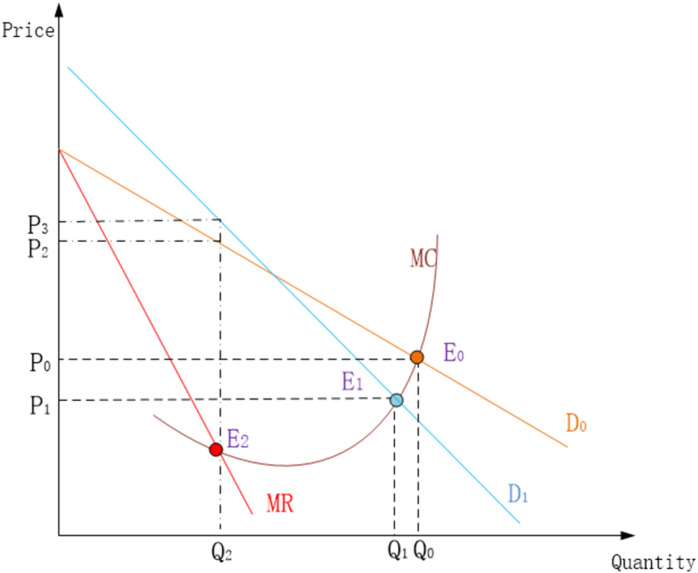
The equilibrium analysis under freight rates regulation.


Fig 1 illustrates why regulation fails. To interpret this figure, we differentiate the demand curves of mass freight (the cargo has high price elasticity, differentiated by China Railway Transport Category on http://www.95306.cn) transportation $D_{0}$ and general cargo transportation in order to get revenue. *D*_0_ *Q*_0_. and $D_{1}Q_{1}$. Combined with the utility function of the consumer U(Q) and production function C(Q), we establish consumer surplus and producer surplus as follows:

$$CS:U(Q)-D_{0}Q_{0}-D_{1}Q_{1}$$ (1)

$$CS:U(Q)-D_{0}Q_{0}-D_{1}Q_{1}$$ (2)

Then the function of social welfares is denoted by


W(Q)=CS+PS=U(Q)−C(Q) (3)

The first derivative condition is obtained easily as the following


$$\frac{dW(Q)}{dQ_{i}}=D_{i}(Q_{i})-MC=0i\in (0,1)$$ (4)

Solving this Eq (4) will lead to a single solution in which the equilibrium point $E_{0}(P_{0},Q_{0})$ and $E_{1}(P_{1},Q_{1})$ of government expectation are obtained. However, the CRC wants to find its optimal production schedule $E_{2}$: the profit-maximizing output will come where the marginal revenue curve (MR) intersects the marginal cost curve (MC).

As shown in Fig 1, the optimal equilibrium price $\left(P_{2},P_{3}\right)$ that CRC wants is higher than the regulated price $\left(P_{0},P_{1}\right)$ of equilibrium point. Owing to regulation, CRC cannot use price leverage to adjust market demand, maximize self-interests. Nonetheless, they will consciously control freight volumes $Q_{2}$ where can maximize their own interests, instead of expanding to the point $\left(Q_{0},Q_{1}\right)$ that government is looking forward to.

### 2.2. Regulation problem description

The regulation will exacerbate shortages of capacity supply in areas with limited transport capacity. Hence, the CRC has the right to allocate the insufficient transport capacity. Transportation plan distribution permissions can be abused by related departments and staff for personal gain, resulting in the corruption phenomenon known as “right rent-seeking.” A number of CRC-affiliated borderline organizations emerge. They provide certain extensibility services and take advantage of the owner’s psychology of being willing to pay extra costs for freight volume, inadvertently raising transportation prices by charging out-of-the-money (additional service income).

Thus, the purpose of the Chinese government’s trying to control the railway freight rates, maximizing social welfare, was not achieved. On the contrary, a few inefficient or even illegal behaviors have occurred. Meanwhile, the existence of railway freight rate control will attract more transportation demands to the railway because it is cheaper, which also aggravates the strain of railway transportation capacities. Therefore, it generates a demand-crowded phenomenon and increases waiting time for loading and unloading, as well as travel time. Furthermore, there is a risk that freight demands will not be met on time.Then, the generalized circulation cost of the shipper is also increasing. Furthermore, the external cost of shipping caused by the lack of transport capacity is challenging to internalize. Those resources cannot serve potential freight demands, resulting in a waste of resources and the loss of social welfare.

Through the above analysis, the performance of railway cargo transportation is ultimately limited by freight rate regulation. It is generally believed that regulating activity has disadvantages. The regulation would be inefficient and should be reformed as soon as possible. However, it will be difficult to find the new equilibrium point between social welfare and enterprise benefits in the process of reform. As a result, this paper may contribute to research on the impact of macroeconomic policy reform on economic variables. In addition, our research will also have some implications for the Chinese government’s decision on policy adjustments in the railway industry in the future.

### 2.3. Research motivation

As mentioned earlier, short-term research is more challenging than long-term economic research, but more practical for governments [33], especially railway enterprises undergoing regulation easing. In a regulated period, the government sets a ceiling or conducts an approval process to regulate the price of rail freight. Once the government deregulates the railway freight rates, railway enterprises will adopt new price competition strategies based on their own judgment of the market, leading to fluctuations in freight rates. The short-term pressure on revenues and profits of railway enterprises will be present. In addition, excessive deregulation or complete abolition of controls could significantly impact the railroad transportation market in the short term, necessitating adjustments from stakeholders and the government.

Short-term economic impacts are the initial stage of long-term economic impacts, which accumulate and deepen over time, ultimately leading to long-term economic consequences. However, there is also a relationship of interaction and feedback between short-term and long-term economic impacts. For example, certain short-term government policies may have an impact on the railroad freight market, and these short-term impacts may prompt market players to take corresponding strategies and actions. These actions may further affect the structure of the market and the competitive landscape, thereby having an impact on the long-term economy. Thus, the short-term economic impacts should not be overlooked.

**Fig 2:**
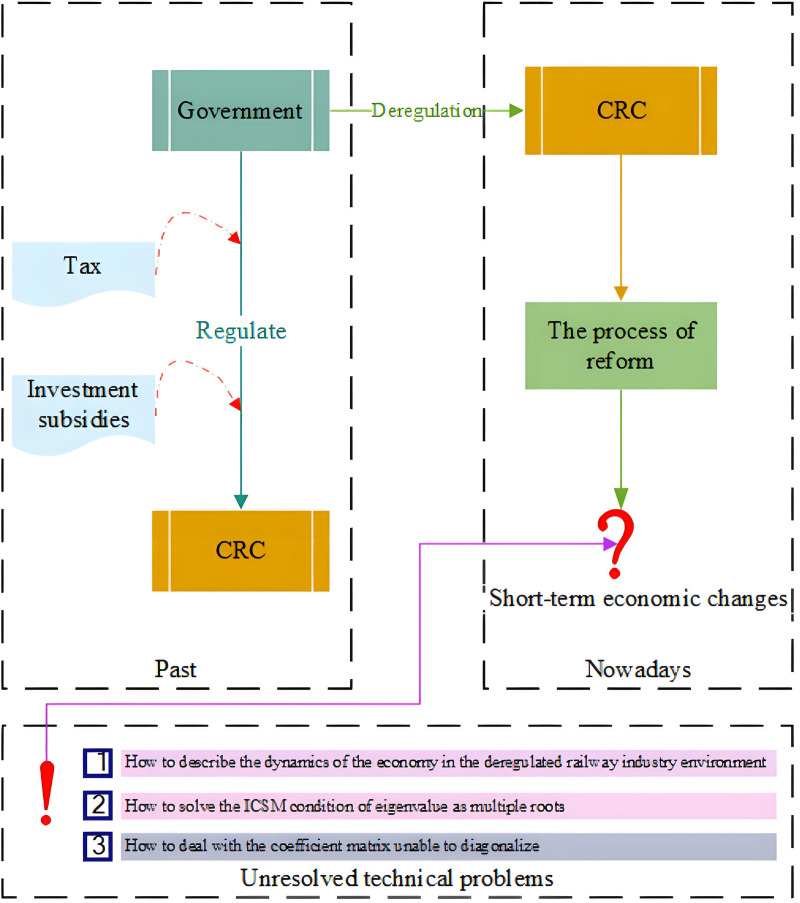
The motivation framework.

However, limited to existing research methodologies, there are still some unresolved technical issues to be dealt with in the study of short-run economic change, which is why this paper is motivated (Fig 2).

## 3. Methodology

In this section, we formulate the methodological framework of this study. First, the objective function is described. Second, a basic multidimensional differential dynamical system and related assumptions are introduced. Finally, in accordance with the current situation, a new improved model containing four ordinary differential equations for the transportation industry is discussed.

### 3.1. Objective function description

Manufactured goods and agricultural products were produced in all areas of the country in the past and had to be shipped great distances for national consumption. The adoption of freight rate controls by governments has facilitated the transnational flow of production factors and goods. Section II elaborates on the fact that freight rate control is unreasonable in theory and in practice, emphasizing the need for regulatory relaxation. Predictably, maximizing business enterprise profits is the objective of CRC once the regulations are relaxed.

**Fig 3:**
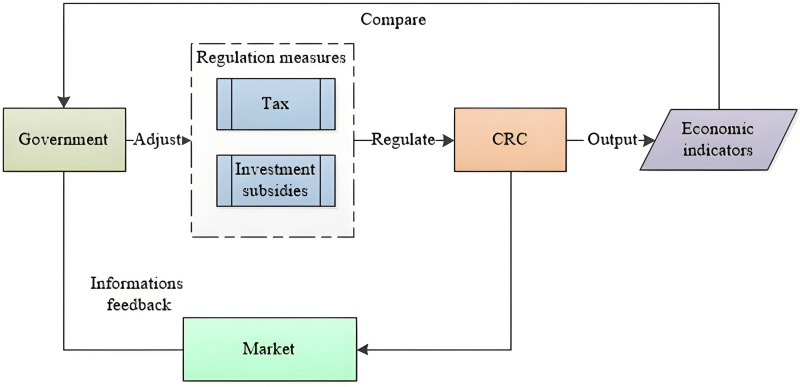
The flowchart of economic control on CRC.

Deregulation allows transportation companies to operate freely with minimal government interference, but it is important to establish market-based regulation to prevent abuse of power. A bigger worry is the market for government, in which the CRC would enjoy a monopoly in China. Thus, to keep CRC from becoming a monopoly, the government tends to use two major macroeconomic-based regulatory measures: tax controls and investment subsidies, as shown in Fig 3. Therefore, the objectives of the model are determined by: revenue from providing transportation services; welfare loss under a given price due to the existence of regulation of negative externalities; and operating cost.

Table 1 provides a list of all notations used in our work to facilitate comprehension of the model. Certain symbols will be explained in greater depth as they appear in later sections of this paper.

**Table 1:** Summary of notations.

Notation	descriptions
*p*	*freight rates*
*q*	*freight volumes*
*l*	*labor force*
*σ*	*tax rate*
*π*	*investment subsidies rate*
*U(p,q)*	*transportation revenue*
σu(p)	*welfares loss*
*C(l)*	*labor cost function*
*t*	*time*
$\dot{p}(t)$	*derivative of p with respect to t*
$\dot{q}(t)$	*derivative of q with respect to t*
*ϕ*, *φ*	*control variables*
*δ*	*constant rate of time preference*
*λ*	*undetermined parameter*
$h_{\sigma }$, $h_{\pi }$	*constant functions of time*
*ε*	*coefficient of variation scale of policies reform*
*A*	*coefficient matrix*
$\upsilon_{i}$	*positive eigenvalues*
$\mu_{i}$	*negative eigenvalues*
*V*	*invertible matrix*
*J*	*Jordon matrix*
H(−)	*Laplace transform*

### 3.2. Basic model building

The revenue that CRC earns by providing transportation services allows enterprises to recoup the costs of operation and labor. In this way, CRC wants to make as much money as possible, whether or not it needs to be regulated. Therefore, the objective of the comparative static model is to determine, based on the principle of utility maximization of CRC. The formulation of the basic model which is considered as the following extension of Wirl’s [34] framework is shown in Eq (5).


$$\underset{p,q}{MAXW}=\int_{0}^{\infty }\Gamma (u(p),q,l)e^{-\delta t}dt$$

$$=\int_{0}^{\infty }[U(p,q)-\sigma u(p)-C(l)]e^{-\delta t}dt$$ (5)

subject to the given initial conditions *p(0) = p*_*0*_*, q(0) = q*_*0*_, and


$$\dot{p}(t)=l(t)$$ (6)

$$\dot{q}(t)=\alpha (q(t))-p(t)$$ (7)

where Eqs (6 and 7) are continuously differentiable functions, p(t) and q(t) stand for state variables of freight rates and volumes, respectively. In scientific experiments, the control variable is used as a standard of comparison that does not change. δ(0<δ<1) stands for the constant rate of time preference. Eq (6) indicates that the freight rate is positively related to the labor force. It’s because CRC needs freight income to pay for the spending. And more labor is a sign of lower labor productivity in the industry, which means CRC needs to pay higher labor costs. Eq (7) indicates that there is a negative correlation between the gradient of freight volumes and rates instead of a simple linear relationship. That means higher freight rates reduce volumes and vice versa.

In the objective function Eq (5), CRC gets the income U(p,q) from providing the quantity supplied *q* (freight volumes) at a price of *p* (freight rates). For convenience, we denote U(p,q)=u(p)q, u(p)=p, and u"(p)≤0. σu(p) stands for welfares loss under a given price due to the existence of regulation of negative externalities. If deregulation occurs, it would also cause social welfare loss owing to the excess profits transportation enterprises would obtain. C(l) stands for the cost function when the labor force is *l*. We assume it is a monotone increasing convex function and approximately equal to *CS.* Hence, the reasonable cost function based labor force is as the following:


$$C(l)=l+\frac{1}{2}\lambda l^{2}-\pi l$$ (8)

α(q(t))=q(q−1) (9)

where *λ* stand for the parameters to be determined.

We assume that the marginal value of freight rates and volumes are ϕ(t) and φ(t) respectively, and according to optimization theory, we will get the Euler equations as the following:


$$\dot{p}(t)=(\pi +\phi -1)/\lambda$$ (10)

$$\dot{q}(t)=\alpha (q(t))-p$$ (11)

$$\dot{\phi }(t)=\delta \phi -u(p)+\varphi$$ (12)

$$\dot{\varphi }(t)=(\delta -\alpha (q))\varphi +\sigma$$ (13)

with the boundary conditions limt→∞p(t)<∞, limt→∞q(t)<∞, limt→∞ϕ(t)<∞, limt→∞φ(t)<∞, and $p(0)=p_{0},q(0)=q_{0}$.

By combining Eqs (10–13), we obtain the equilibrium point $\left(p^{*},q^{*},\phi^{*},\varphi^{*}\right)$ easily through calculation when $\dot{p}(t)=\dot{q}(t)=\dot{\phi }(t)=\dot{\varphi }(t)=0$, as defined in Eqs (14a–14d)


$$p^{*}=q^{*}(q^{*}-1)$$ (14a)

$$q^{*}=\frac{1}{2}(1+\delta +\frac{\sigma }{\delta -\delta \pi -u(p)})$$ (14b)

$$\varphi^{*}=u(p)-\delta \phi^{*}$$ (14c)

$$\phi^{*}=1-\pi$$ (14d)

In order to make the comparative static model for the short-term economic influence analysis, which is explained in the next section, we first make the following assumptions:

#### Assumption 1.

Considering that the analysis of general cargo transportation in this paper will be more meaningful and effective in adjusting deregulation policies. Therefore, the object of this paper is general cargo transportation that is easy to market-oriented.

#### Assumption 2.

Step function $h_{i}(t)$ is bounded, and convergences to constant functions, ∀i=1,⋯,i..

#### Assumption 3.

Considering that the objective of this research is to analyze the short-term economic influence after deregulation, we consider the government is expected to use tax controls and investment subsidies for macro-control after deregulation.

### 3.3. Model improving

As we know, the tax system is different in China than in the Occident. In China, as one administrative measure, a taxation model based on turnover tax (an indirect tax) is implemented. The taxation model is not intended to encourage competition and free trade but to promote the development of state-owned enterprises. As a state-owned enterprise, CRC is still supported by the state finance from investment construction to operation. Therefore, both tax policies and investment subsidies are the government’s common control measures for state-owned enterprises [35].

In accordance with the actual situation, we suppose that the government has been taxing at a constant rate *σ*, granting investment subsidies at a constant rate before deregulation in time t=0. When the deregulation policies are announced at that time *t*, *σ* and *π* will change by $\varepsilon h_{\sigma }(t)$ and $\varepsilon h_{\pi }(t)$, respectively. where $h_{\sigma }$ and $h_{\pi }$ are eventually constant functions of time, *ε* stands for the coefficient of variation scale of policy reform.

Through the above assumptions and analysis, update Eqs (10–13), the further developed Euler equations for the deregulation of railway cargo transportation, are obtained separately as the following:


$$\dot{p}(t)=(\pi +\phi +\varepsilon h_{\pi }(t)-1)/\lambda$$ (15a)

$$\dot{q}(t)=\alpha (q(t))-p$$ (15b)

$$\dot{\phi }(t)=\delta \phi -u(p)+\varphi$$ (15c)

$$\dot{\varphi }(t)=(\delta -\alpha '(q))\varphi +\sigma +\varepsilon h_{\sigma }(t)$$ (15d)

We denote p(t,ε), q(t,ε), ϕ(t,ε), φ(t,ε) as the solutions of further developed Euler equations. The objective of this study is to analyze the short-term economic influence on initial variables after deregulation. We convert it into a mathematical problem how to find the effects of *ε* on endogenous variables at future times, $p_{\varepsilon }(t)$, $q_{\varepsilon }(t)$, $\phi_{\varepsilon }(t)$ and $\varphi_{\varepsilon }(t)$. Therefore, differentiating Eqs (15a–15d) with respect to the parameters, the linearized economic system is obtained as follows:


$$\left[\begin{array}{l} {\dot{p}}_{\varepsilon }(t)\\ {\dot{q}}_{\varepsilon }(t)\\ {\dot{\phi }}_{\varepsilon }(t)\\ {\dot{\varphi }}_{\varepsilon }(t) \end{array}\right]=A\left[\begin{array}{l} p_{\varepsilon }(t)\\ q_{\varepsilon }(t)\\ \phi_{\varepsilon }(t)\\ \varphi_{\varepsilon }(t) \end{array}\right]+Bh_{\varepsilon }(t)=\left[\begin{array}{cccc} 0 & 0 & 1/\lambda & 0\\ -1 & \alpha^{\prime } & 0 & 0\\ 0 & 0 & \delta & 1\\ 0 & 2\varphi & 0 & \delta -\alpha^{\prime } \end{array}\right]\left[\begin{array}{l} p_{\varepsilon }(t)\\ q_{\varepsilon }(t)\\ \phi_{\varepsilon }(t)\\ \varphi_{\varepsilon }(t) \end{array}\right]+\left[\begin{array}{cc} 0 & 1/\lambda \\ 0 & 0\\ 0 & 0\\ 1 & 0 \end{array}\right]h_{\varepsilon }(t)$$ (16)

**Fig 4:**
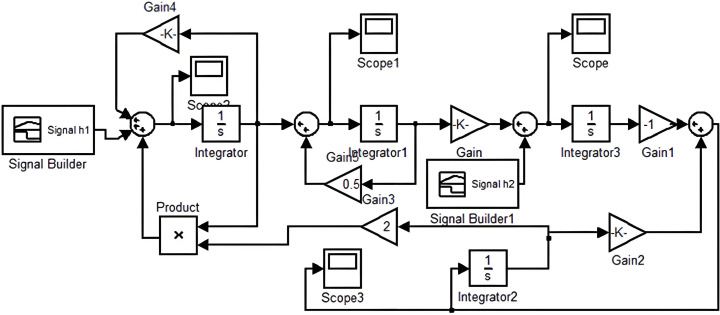
The evaluation flow.

In which: *A* is a n×n constant matrix and *B* is a n×i constant matrix. The coefficient matrix *A* is the Jacobian matrix of the vector function, it has $n_{1}$ distinct negative eigenvalues $\upsilon_{1},\cdots ,\upsilon_{n_{1}}$, and $n-n_{1}$ distinct positive eigenvalues $\mu_{1},\cdots ,\mu_{n-n_{1}}$. Fig 4 shows the simple diagram of linearized economic system.

According to matrix operation rules, the coefficient matrix *A* in Eq (16) can be diagonalized by an invertible matrix *V* and a Jordon matrix *J* as the following:


$$A=VJV^{-1}$$ (17)

In a coefficient matrix *A*, the number of eigenvalues with a negative real part is the same as the number of predetermined variables (state variables). It’s very common to denote the two positive eigenvalues of *A* by $\upsilon_{1}$ and $\upsilon_{2}$, the two negative eigenvalues of *A* by $\mu_{1}$ and $\mu_{2}$. Considering that one of the objectives of this paper is to solve the defect of eigenvalues as multiple roots, it is assumed that the eigenvalues $\mu_{1}=\mu_{2}=\mu >0$. Therefore, the Jordan matrix *J* can be expressed as the following:


$$J=\left[\begin{array}{cccc} \upsilon_{1} & \psi & 0 & 0\\ 0 & \upsilon_{2} & 0 & 0\\ 0 & 0 & \mu & 1\\ 0 & 0 & 0 & \mu \end{array}\right]$$

$$Where\psi = \{\begin{array}{l} 1,if\upsilon_{1}=\upsilon_{2}\\ 0,otherwise \end{array}.$$

Then substituting Eq (17) into Eq (16) and left multiplying by *V*, it is easy to obtain the update Eq (18) as the following:


$$V^{-1}\left[\begin{array}{l} \dot{p}(t)\\ \dot{q}(t)\\ \dot{\phi }(t)\\ \dot{\varphi }(t) \end{array}\right]=J\left[\begin{array}{l} p_{\varepsilon }(t)\\ q_{\varepsilon }(t)\\ \phi_{\varepsilon }(t)\\ \varphi_{\varepsilon }(t) \end{array}\right]+V^{-1}Bh_{\varepsilon }(t)$$ (18)

In which: B=[0,0,0,1;1/λ,0,0,0]

For convenience, we denote $w_{\varepsilon }={\left(w_{\varepsilon 1},w_{\varepsilon 2},w_{\varepsilon 3},w_{\varepsilon 4}\right)}^{T}=V^{-1}{\left(p_{\varepsilon },q_{\varepsilon },\phi_{\varepsilon },\varphi_{\varepsilon }\right)}^{T}$ and $\beta_{i}=V^{-1}Bh_{\varepsilon }(t)={\left(\beta_{1},\beta_{2},\beta_{3},\beta_{4}\right)}^{T}$, substituting matrix *B* into $\beta_{i}$, we obtain Eq (19) as the following:


$$\beta_{i}={\left(V^{-1}\right)}_{i4}h_{\sigma }(t)+\frac{1}{\lambda }{\left(V^{-1}\right)}_{i1}h_{\pi }(t)$$ (19)

where i=1,2,3,4, and ${\left(V^{-1}\right)}_{m\times n}$ is the elements in matrix $V^{-1}$ of row and column *n*.

**Fig 5:**
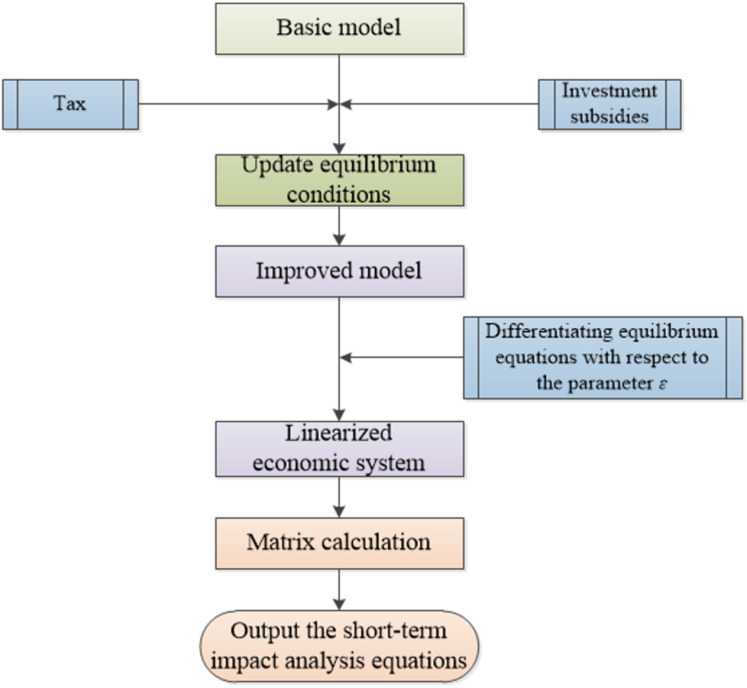
The comprehensive evaluation of cost factor.


As shown in Fig 5, through the above calculations and derivations, the equations for the short-term economic influence analysis are obtained in accordance with the ICSM as the following:

$${\dot{w}}_{\varepsilon i}=Jw_{\varepsilon i}+\beta_{i}$$ (20)

## 4. Solution approaches

The system contains four ordinary differential equations. It easily gets results by solving first-order ordinary differential equations with the uniqueness satisfying the conditions on the eigenvalues. If the eigenvalues of the coefficient matrix are multiple roots, the equations cannot be directly solved. Thus, a new method using the recursive principle and Laplace transform that solves the condition of eigenvalue as multiple roots and coefficient matrix unable to diagonalize is proposed as the following:

**Step 1** Initialization of the computation.

In accordance with the deregulation process that rail transport is undergoing, a set of parameters are considered that should be used to calculate the coefficient matrix *A*. The eigenvalues of the coefficient matrix *A* can be calculated.

**Step 2** Calculate $w_{\varepsilon 4}(t)$ in Eq (20)

The last equation in Eq (20) can be directly calculated according to the features of the Jordon matrix *J* as the following:


$$\begin{array}{l} w_{\varepsilon 4}(t)=-e^{\mu t}\int_{t}^{\infty }\beta_{4}(x)e^{-\mu t}dx\equiv -e^{\mu t}Z_{4}(t)\\ =-e^{\mu t}\left[{\left(V^{-1}\right)}_{44}H_{\sigma t}(\mu )+\frac{1}{\lambda }{\left(V^{-1}\right)}_{41}H_{\pi t}(\mu )\right] \end{array}$$ (21)

where $H_{\sigma t}(\mu )$ and $H_{\pi t}(\mu )$ are the Laplace transform of $h_{\sigma }(x)$ and $h_{\pi }(x)$.

**Step 3** Calculate $w_{\varepsilon 3}(t)$ in Eq (20)

In this step, we substitute $w_{\varepsilon 4}(t)$ into Eq (20), it is easy to calculate the $w_{\varepsilon 3}(t)$ base for recursive methods as the following:


$$\begin{array}{l} w_{\varepsilon 3}(t)=e^{\mu t} [{\left(V^{-1}\right)}_{44}{\bar{H}}_{\sigma t}(\mu )+\frac{1}{\lambda }{\left(V^{-1}\right)}_{41}{\bar{H}}_{\pi t}(\mu )\\ -\left.{\left(V^{-1}\right)}_{34}H_{\sigma t}(\mu )+\frac{1}{\lambda }{\left(V^{-1}\right)}_{31}H_{\pi t}(\mu )\right] \end{array}$$ (22)

where ${\bar{H}}_{\sigma t}(\mu )$ and ${\bar{H}}_{\pi t}(\mu )$ are the Laplace transform of $H_{\sigma t}(\mu )$ and $H_{\pi t}(\mu )$. $p_{\varepsilon }(t)$ and $q_{\varepsilon }(t)$ are state variables that cannot jump initially, thus $p_{\varepsilon }(0)=q_{\varepsilon }(0)=0$.

**Step 4** Calculate the other equations in the system

In this step, $w_{\varepsilon 1}(0)$, $w_{\varepsilon 2}(0)$, $\phi_{\varepsilon }(0)$ and $\varphi_{\varepsilon }(0)$ are calculated in a similar manner, This problem can be transferred to the following system of equation groups problem. We can calculate the $w_{\varepsilon 1}(0)$, $w_{\varepsilon 2}(0)$, $\phi_{\varepsilon }(0)$ and $\varphi_{\varepsilon }(0)$ by solving the following system of equation groups:


$$\left[\begin{array}{cccc} {\left(V^{-1}\right)}_{11} & {\left(V^{-1}\right)}_{12} & {\left(V^{-1}\right)}_{13} & {\left(V^{-1}\right)}_{14}\\ {\left(V^{-1}\right)}_{21} & {\left(V^{-1}\right)}_{22} & {\left(V^{-1}\right)}_{23} & {\left(V^{-1}\right)}_{24}\\ {\left(V^{-1}\right)}_{31} & {\left(V^{-1}\right)}_{32} & {\left(V^{-1}\right)}_{33} & {\left(V^{-1}\right)}_{34}\\ {\left(V^{-1}\right)}_{41} & {\left(V^{-1}\right)}_{42} & {\left(V^{-1}\right)}_{43} & {\left(V^{-1}\right)}_{44} \end{array}\right]\left[\begin{array}{c} 0\\ 0\\ \phi_{\varepsilon }(0)\\ \varphi_{\varepsilon }(0) \end{array}\right]=\left[\begin{array}{c} w_{\varepsilon 1}(0)\\ w_{\varepsilon 2}(0)\\ {\bar{Ζ}}_{4}(0)-{Ζ}_{3}(0)\\ -{Ζ}_{4}(0) \end{array}\right]$$ (23)

**Step 5** Calculate the short-term economic influence after deregulation

By combining Eq (18) with the above calculations, the macroeconomic policies that influence the economic variables of CRC in the short-term are represented as the following:


p˙ε(0)=[ϕε(0)+hε(0)]λ (24a)

$${\dot{q}}_{\varepsilon }=0$$ (24b)

$${\dot{\phi }}_{\varepsilon }(0)=\delta \phi_{\varepsilon }(0)+\varphi_{\varepsilon }(0)$$ (24c)

$${\dot{\varphi }}_{\varepsilon }(0)=(\delta -\alpha '(q^{*}))\varphi_{\varepsilon }+h_{\varepsilon }(0)$$ (24d)

Furthermore, in accordance with the further developed Euler Eqs (15a–15d), the policy reform influence on the labor force in the short-term is calculated as follows:


$$l(t)=(\pi +\phi +\varepsilon h_{\pi }(t)-1)/\lambda$$ (25)

$$l_{\varepsilon }(0)=\left\{\phi_{\varepsilon }(0)+h_{\pi }(0)\right\}/\lambda$$ (26)

$${\dot{l}}_{\varepsilon }(0)=\left\{{\dot{\phi }}_{\varepsilon }(0)+{\dot{h}}_{\pi }(0)\right\}/\lambda$$ (27)

Through the above five steps and the results from Eq (24b), this article finds an interesting conclusion that government policy reform has no direct impact on the freight rates and the stock of freight volumes, which are determined by the state variables that cannot be changed in the model. In real transportation activities, it can be understood as the following: The lower freight rates inevitably increase the freight volumes demanded is subject, however, to the saturated freight volumes on railway lines, which are in short supply. That is why it is so difficult to increase new freight volumes in the short term. In order to discuss the short-term economic influence intuitively, a case study is given in the next section.

## 5. Case study and result discussion

### 5.1. Data settings and calculates

This section discusses the application results of the methodology. Table 2 contains the parameter values for numerical analysis, which are derived from the literature and official websites. These include the constant discount rate and utility of price. Meanwhile, the constant discount rate and investment subsidy rate are obtained based on the report of market prospects and investment strategy planning for the China railway industry (2020-2025) [36].

**Table 2:** Parameter values for numerical analysis.

Parameter	Value
Constant rate of time preference *δ*	0.5
Taxing rate *σ*	30%
Investment subsidies rate *π*	10%
Utility of price u(p)	1

In accordance with the optimality conditions Eqs (14a–14d), the coefficient matrix can be calculated and expressed as follows:


A=001λ0−1−1220000121000611

For the coefficient matrix, the eigenvalues should satisfy the following equation.


det(A−υI)=0 (28)

The solutions of the characteristic equation should satisfy the equation as follows.


$$\upsilon^{4}-tr(J)\upsilon^{3}+B\upsilon^{2}-C\upsilon +\det(J)=0$$ (29)

Then calculate the eigenvalues $\upsilon_{i}$ as follows.


$$\upsilon_{i}=\frac{r}{2}\pm \sqrt{{\left(\frac{r}{2}\right)}^{2}-\frac{K}{2}\pm \frac{1}{2}\sqrt{K^{2}-4\det(J)}}$$ (30)

in which: $r=\frac{1}{2}$, $K=B-r^{2}$, $\det(J)=\frac{11}{10\lambda }$, $B=\frac{109}{484}$, $C=\frac{3}{242}$.

Furthermore, we get two negative eigenvalues $\upsilon_{1}$ = $\upsilon_{2}$ = 0.5237, also two positive eigenvalues $\mu_{1}$ = $\mu_{2}$ =-0.0237.

Then substituting eigenvalues $\upsilon_{i}$ into Eqs (24a–27), it is easy to obtain the Eq (31) as the following:


$$\begin{array}{l} l_{\varepsilon }(0)=\text{1}.3971e-004*h_{\pi }(0)+0.1309*{\bar{H}}_{\sigma t}(\mu )-\text{6}.7511e-005*{\bar{H}}_{\pi t}(\mu )\\ -\text{3}.7581e-010*H_{\sigma t}(\mu )+\text{6}.7636e-005*H_{\pi t}(\mu ) \end{array}$$ (31)

### 5.2. Results analysis

#### 5.2.1. Government investment subsidies impact on freight in the short-term.

In order to study the government’s investment subsidies influence on freight, this subsection assumes that the government only implements control through investment subsidies, there is no change in the tax policies. Then we have


$$\begin{array}{l} h_{\pi }(t)=e^{-\mu t}\\ h_{\sigma }(t)=0 \end{array}$$ (32)

Moreover, using the Laplace transform, the relevant parameters can be calculated as the following:


$$\begin{array}{l} H_{\pi t}(\mu )=0.9547\\ {\bar{H}}_{\pi t}(\mu )=0.9114\\ H_{\sigma t}(\mu )={\bar{H}}_{\sigma t}(\mu )=0 \end{array}$$ (33)

Furthermore, if we substitute Eqs (32 and 33) into Eq (31), the investment subsidies influence on the freight rates and labor force in the short term is calculated as follows:


$${\dot{p}}_{\varepsilon }(0)=l_{\varepsilon }(0)=1.4275\text{e-004}$$ (32)

In general, the impact of government investment subsidies on rail strategy is a complex issue involving multiple factors and stakeholders. Existing studies agree that substantial government subsidies can attract more passengers and increase transport volume by reducing freight rates for railway companies. Investment subsidies also encourage operational efficiency and better services, such as purchasing new equipment, improving maintenance programs, and increasing train frequency. However, our research differs significantly from the conventional understanding of scholars. The results indicate that increasing investment subsidies can improve labor employment and freight rates. That is mainly because the investment subsidies will be conducive to the railway constructing railway infrastructure that can provide more employment positions. However, the freight rates need to be readjusted at the given costs in order to ensure recovery of the investment as soon as possible. The investment and construction of China High-Speed Railway (CHR) is an obvious instance. For one thing, investment subsidies will promote HSR construction and provide more jobs. Another thing is that HSR will charge higher fees that can cover the costs of operations and investment.

In addition, the data results show that government investment subsidies have a relatively small impact on both labor employment and freight rates, primarily due to various reasons as described below.

1. Freight rates are primarily determined by market supply and demand, and if government subsidies are insufficient to alter these relationships, their impact on freight rates will be minimal.
2. Government subsidies may not be evenly distributed. If the subsidy is mainly concentrated in some specific transportation enterprises, such as China Railway, then its impact on the overall level of labor employment and freight rates may not be significant.
3. Government investment subsidies can encourage railway enterprises to enhance operational efficiency, thereby reducing transportation costs. In this case, the subsidy indirectly impacts both labor employment and freight rates by enhancing efficiency rather than directly reducing them.
4. If there is sufficient competition in the transportation market, it is difficult for individual enterprises or government subsidies to have a significant impact on labor employment and freight rates. Competition will motivate firms to provide better quality services and more reasonable prices.

In short, the impact of government investment subsidies on labor employment and freight rates depends on a variety of factors, including the size of the subsidy, the distribution method, the market environment, and the competition situation. In practice, the government and enterprises need to take these factors into account and develop a reasonable subsidy policy to achieve anticipated economic and social benefits.

#### 5.2.2. Government tax impacts on freight in the short-term.

In order to study the tax’s influence on freight, this subsection assumes that the government implements control through taxation. There is no government subsidy for investment. Then we have


$$\begin{array}{l} h_{\sigma }(t)=e^{-\mu t}\\ h_{\pi }(t)=0 \end{array}$$ (35)

Moreover, using the Laplace transform, the relevant parameters can be calculated as the following:


$$\begin{array}{l} H_{\sigma t}(\mu )=0.9547\\ {\bar{H}}_{\sigma t}(\mu )=0.9114\\ H_{\pi t}(\mu )={\bar{H}}_{\pi t}(\mu )=0 \end{array}$$ (36)

Furthermore, if we substitute Eqs (32 and 33) into Eq (31), the tax policy influence on the freight rates and labor force in the short term is calculated as follows:


$${\dot{p}}_{\varepsilon }(0)=l_{\varepsilon }(0)=0.1193$$ (37)

Although the tax policy has no direct influence on the freight rates and the stock of freight volumes, it is positively related to the freight rates. Tax policies can significantly impact freight rates by adjusting tax rates and types. Increased taxes can increase transportation costs, while reduced taxes can decrease them. Tax policies can impact the labor market by increasing supply and demand through tax breaks, and by affecting the flow and distribution of labor by differentiating tax collection for workers in different industries or income levels.In essence, tax policies play a crucial role in shaping the overall economic landscape. Specifically for railway transportation enterprises, if the government adopts increased taxes on CRC, it will transfer taxes to consumers through price increases in the case of deregulation. Besides, the contradiction between supply and demand will result in freight rates rising. Furthermore, the reforms strengthen the enterprise attribute of CRC, with management decisions based on whether they enhance labor productivity, profit, or other income. Then, CRC will be inclined to hire excellent technical personnel by offering a very competitive salary in all positions.

As the results show, after deregulation, tax policy is more sensitive to the impact on prices than investment subsidies in the short term.The former tends to be 0.1193 more than the latter. There are several main reasons why tax policy has a greater impact on labor employment and freight rates than government investment subsidies:

1. Tax policy is universally applicable, impacting all market players, including producers and consumers, who must pay various taxes and fees, making its impact more extensive and profound. Meanwhile, tax policy directly impacts market behavior, such as increasing consumer purchase costs through consumption tax on commodities. This direct influence leads to a rapid and significant effect on freight rates and labor demand.
2. Tax policy is a flexible approach where the government can adjust tax rates, bases, and incentives based on economic conditions and policy objectives. This allows it to better adapt to market changes, impacting market prices and labor demand. Tax policy is often viewed as a fair method of income distribution, aiming to reduce the wealth gap and promote social equity by imposing different taxes on different income groups. In macroeconomic terms, these effects are not directly regulated by investment subsidies.
3. The management and operation of China’s railways have overemphasized public welfare in the past. The government takes railway freight as a necessary condition to valorize for a long time. It is difficult for the railway industry to attract investment funds as a result of the decreasing investment enthusiasm of fund holders. That is another reason why variables are less sensitive to investment subsidies than tax policies in the short-term.

Furthermore, we can calculate the impact of policy reform on social welfare. Taking Laplace transform for Eq (18) and letting $P_{\varepsilon }(s),Q_{\varepsilon }(s),\Phi_{\varepsilon }(s),\Psi_{\varepsilon }(s)$ be the Laplace transforms of $p_{\varepsilon }(t),q_{\varepsilon }(t),\phi_{\varepsilon }(t),\varphi_{\varepsilon }(t)$ with parameter respectively, we have


$$\left[\begin{array}{l} P_{\varepsilon }(s)\\ Q_{\varepsilon }(s)\\ \Phi_{\varepsilon }(s)\\ \Psi_{\varepsilon }(s) \end{array}\right]={\left(sI-A\right)}^{-1}\left[BH(s)+\left[\begin{array}{l} \;0\\ \;0\\ \phi_{\varepsilon }(0)\\ \varphi_{\varepsilon }(0) \end{array}\right]\right]$$ (38)

Thus, social welfare change can be represented by the following:


$$\frac{dW}{d\varepsilon }={\left[\begin{array}{l} \nabla_{p}\Gamma \\ \nabla_{q}\Gamma \\ \nabla_{\phi }\Gamma \\ \nabla_{\varphi }\Gamma \end{array}\right]}^{T}\int_{0}^{\infty }e^{-\delta t}\left[\begin{array}{l} p(t)\\ q(t)\\ \phi (t)\\ \varphi (t) \end{array}\right]={\left[\begin{array}{l} \nabla_{p}\Gamma \\ \nabla_{q}\Gamma \\ \nabla_{\phi }\Gamma \\ \nabla_{\varphi }\Gamma \end{array}\right]}^{T}\left[\begin{array}{l} P_{\varepsilon }(\delta )\\ Q_{\varepsilon }(\delta )\\ \Phi_{\varepsilon }(\delta )\\ \Psi_{\varepsilon }(\delta ) \end{array}\right]$$ (39)

where $P_{\varepsilon }(\delta ),Q_{\varepsilon }(\delta ),\Phi_{\varepsilon }(\delta ),\Psi_{\varepsilon }(\delta )$ are defined in Eq (38).

## 6. Conclusions

In this study, we investigated the macroeconomic policies that influence the economic variables of CRC in the short-term. This study has been initiated for various reasons.

1. Short-term economic analysis is challenging due to the complexity and uncertainty of the economic situation, while long-term analysis has a larger time horizon, which can offset the effects of short-term fluctuations, making it easier to analyze and forecast trends.
2. Short-term economic analysis considers government policy adjustments and implementation effects, with policy changes potentially impacting the economic situation. Long-term analysis focuses on market mechanisms and long-term trends, with minimal government policy impact, allowing for a more comprehensive understanding.
3. Short-term economic analysis focuses on economic indicators and variables, which can be challenging to analyze effectively. Long-term analysis, on the other hand, can explore the nature and laws of economic phenomena using methods like time series and regression analysis.

As a result, short-term economic analysis is relatively difficult and requires greater attention to the choice of analytical methods. Considering the characteristics of the railway transportation market, we developed a multidimensional differential dynamical system that contains four ordinary differential equations based on a comparative static model. In order to solve the problems of eigenvalues as multiple roots and coefficient matrices that cannot be diagonalized in ICSM, we devised a new method based on the recursive principle and the Laplace transform.A case based on the real-macroeconomic data of the Chinese railway industry was used to demonstrate the effectiveness of the proposed models and solution approaches. The main conclusions are as follows:

1. Investment subsidies and tax policy have no direct influence on the freight rates and the stock of freight volumes but have been positively related to the freight rates.
2. Tax policy is more sensitive to the impact on prices, which tends to be 0.1193 than investment subsidies in the short term. In practice, the Chinese government should be careful about how it sets tax policy to avoid losing social welfare.
3. An improved solution algorithm intelligently integrates recursive principle and La-place transform to solve ICSM condition of multiple roots and coefficient matrix unable to diagonalize.
4. The method proposed in this article greatly simplifies the analysis process. In addition, it also has scalability that can correspondingly be extended dimensions if the analysis variables increase.

However, this paper only considers the negative externalities brought by railway transportation, but its positive externalities will also be an important factor in the model that needs to be regarded as the derived demand is increasing based on the space dimension. In addition, whether the method can be improved to apply economic analysis in discrete time will be the focus of the next step.
